# Histological Evaluation and Management of Rare Case of Supernumerary “Ghost” Teeth

**DOI:** 10.1155/2017/1965078

**Published:** 2017-05-04

**Authors:** Dino Re, Elena Canciani, Corinne Poli, Laura Buccarella, Marilisa Toma, Andrea Carlo Butti, Claudia Dellavia

**Affiliations:** ^1^University Department, Istituto Stomatologico Italiano and Department of Biomedical, Surgical and Dental Sciences, Università degli Studi di Milano, Milan, Italy; ^2^Department of Biomedical, Surgical and Dental Sciences, Università degli Studi di Milano, Milan, Italy; ^3^Istituto Stomatologico Italiano, Università degli Studi di Milano, Milan, Italy

## Abstract

Supernumerary teeth are teeth that exceed the normal dental formula. Their prevalence in the permanent dentition is 1–14% and they occur more frequently in maxilla with a sex ratio of 2 : 1 in favor of males. They are often associated with syndromes but there are examples of nonsyndromic multiple supernumerary teeth reported in the literature. CBCT is usually the best exam for radiographic diagnosis and treatment planning, because it provides 3D information about location and morphology of supernumerary teeth. This paper reports a rare case of four supernumerary teeth in a nonsyndromic 9-year-old boy. The peculiarity of this case is that two more exceeding teeth were found during surgical procedure. After extraction, all the teeth underwent a histological undecalcified processing for light microscopical examination. The two “ghost” supernumerary teeth seemed to be primordial dental germs, possibly resulting from an altered odontogenic process. After supernumerary teeth extraction, X-rays and exfoliation monitoring are recommended, since permanent retained teeth often erupt naturally or, at least, improve their condition. Radiographic follow-up is also useful in order to assess the formation of further teeth due to the hyperactivity of the dental lamina.

## 1. Introduction

Supernumerary teeth may be defined as any teeth or tooth substance in excess of the usual configuration of primary or permanent dental formula [1]. They are less common in the primary dentition than in the permanent one and males are affected approximately twice than females [2]. According to literature, their prevalence in permanent dentition is 1–14% [3]. Supernumerary teeth occur more frequently in the maxilla (90–98%), especially in the premaxillary region. In fact, the most common exceeding tooth is the so-called “mesiodens”—a tooth that could be found between the two central upper incisors. Other common supernumeraries are, in order of frequency, maxillary fourth molars, maxillary premolars, mandibular premolars, maxillary lateral incisors, mandibular fourth molars, and maxillary premolars [4]. Supernumerary teeth are often associated with syndromes such as the Gardner syndrome, the Crouzon's disease, the Fabry-Anderson syndrome, the Ehler-Danlos syndrome, the Hallermann-Streiff syndrome, and facial fissures or cleidocranial dysplasia [3].

At the present time, the mechanism of their development is not very well understood. The occurrence of multiple supernumerary teeth without any associated syndrome is a very rare phenomenon. Most commonly, only one supernumerary tooth is present in a dentition; less frequently there are two supernumeraries, while three or more supernumerary teeth are rare [4, 5]. The presence of supernumerary teeth could lead to crowding, delayed eruption, diastema, rotation, and resorption of the adjacent teeth; therefore early diagnosis and appropriate treatment are essential [6].

Cone beam computed tomography (CBCT) is considered to be a useful method of evaluation of supernumerary teeth because it provides 3D information about their location and shape [7].

The aim of this paper is to present a rare nonsyndromic case of multiple hyperdontia, with the presence of four supernumerary teeth. The peculiarity of this case is that two supernumerary teeth were not visible neither in the orthopantomography (OPT) nor in the CBCT.

## 2. Case Report

**Figure 1:**
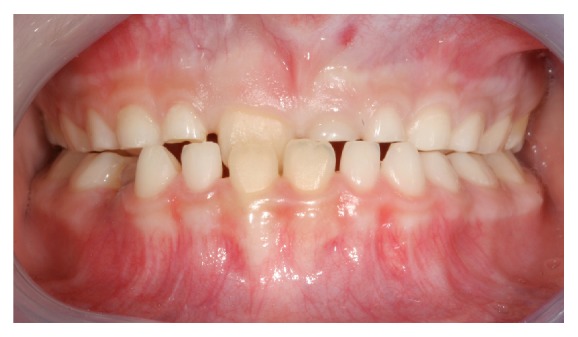
Frontal view of the patient class III occlusion.

We report a case of a hyperdontia in a 9-year-old male patient who underwent a routine dental check-up. At clinical examination, the oral structures appeared normal but he presented a delay of the maxillary left incisor exfoliation (61 as per FDI tooth numbering system) and a delay of 21 eruption. Patient had no symptoms with the teeth involved. The patient presented a negative anamnestic history in his family for altered dentition. Class III molar occlusion was seen with 11 palatally displaced (Figure 1).

**Figure 2:**
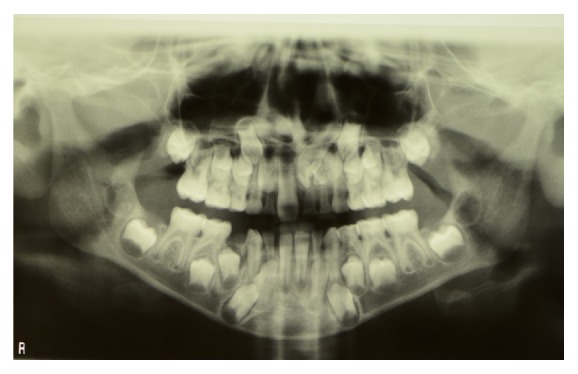
OPT of the patient with supernumerary dental group.

**Figure 3:**
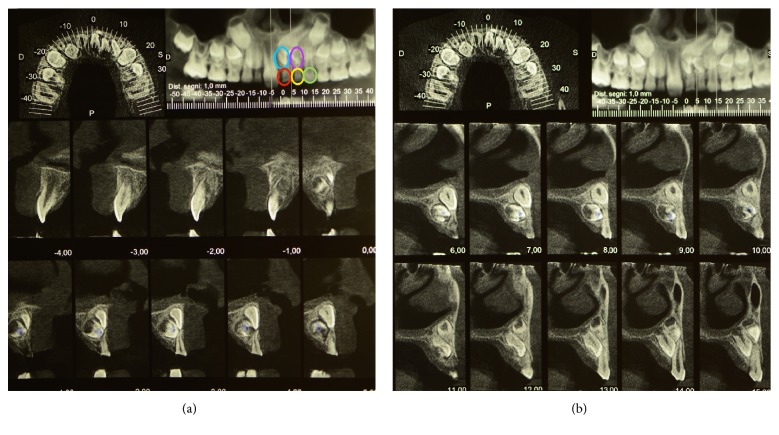
(a) CBCT of the patient showing 3 deciduous teeth (61 in red circle, 62 in yellow circle, and 63 in green circle) and 2 supernumerary teeth (blue and violet circles). (b) CBCT of the patient showing displacement of permanent teeth 21 and 22.

Examination of the OPT (Figure 2) revealed the presence of numerous radiopaque areas in the left incisor region and the consequent difficulty in the eruption of 21 placed over the supernumerary dental group. After acquiring written informed consent, a CBCT was performed and a 3D examination was carried out. The analysis showed a group of 2 supernumerary teeth placed palatally to 61 and 62 with an indefinite shape (Figure 3(a)) and element 21 being displaced apically. Tooth 22 was placed more apically than the contralateral one, palatally and distally in contact with the root of 63 (Figure 3(b)).

A multidisciplinary approach was undertaken, the treatment was decided with an oral surgeon and an orthodontist. The surgical approach involved the extraction of three deciduous teeth 61, 62, and 63 and two supernumerary teeth allowing realignment of 21. A “wait and see” approach was preferred instead of a simultaneous disimpaction of 21 in order to help the natural path of permanent incisor eruption [8–10]. Parents were informed and their consensus was achieved.

**Figure 4:**
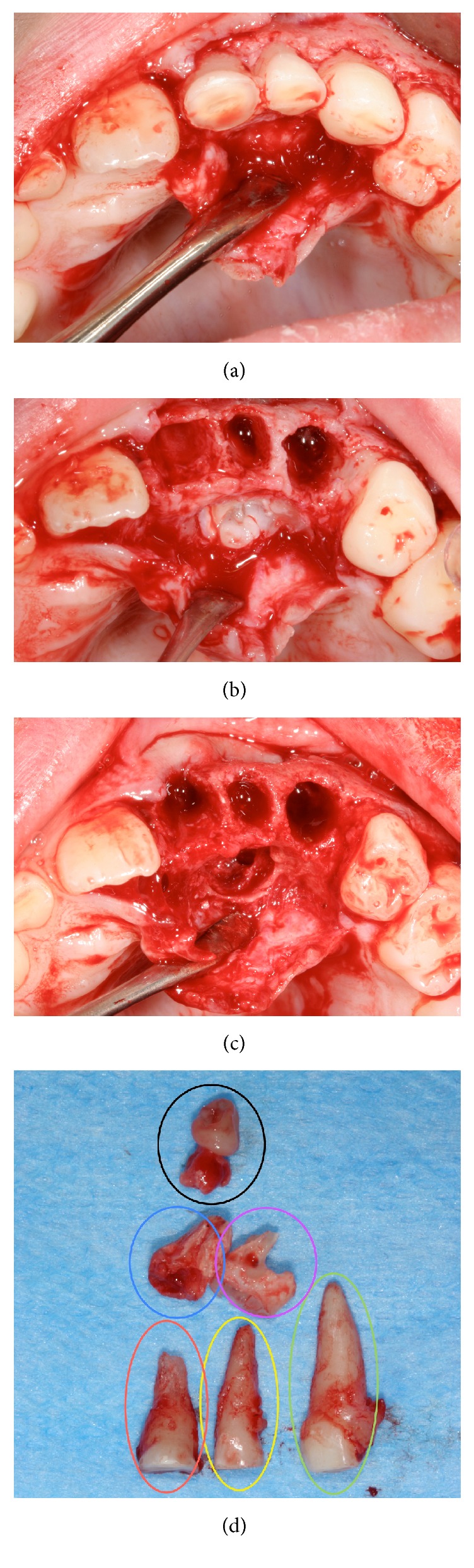
(a) Intraoral view after soft tissue elevation. (b) Intrasurgical view after the extraction of deciduous teeth and the exposure of the supernumeraries. (c) The alveolar site free from supernumerary teeth. (d) Deciduous and supernumerary teeth after the extraction. They are indicated with the same colours used in Figure 3(a). Third and fourth supernumeraries can be seen.

As expected two malformed supernumerary elements were found, but the accurate curettage of the bone revealed a third and fourth abortive elements formed apically and distally to the distal supernumerary tooth (Figures 4(a) and 4(b)). They appeared as small dental germ-like formations with an irregular shape and resulting softer than normal teeth upon compression (Figures 4(c) and 4(d)).

Four supernumerary teeth, two visualized in OPT and CBCT (A—blue circle and B—violet circle as reported in Figures 3(a) and 4(d)) and two discovered upon surgical removal (C and D—black circle in Figure 4(d)), were harvested and processed according to a calcified tissue protocol [11]. In brief, after fixation in formalin 10%/0.1 M phosphate buffer saline solution (pH 7.4), the teeth were dehydrated (70%, 90%, 96%, and 100%), infiltrated in different ratios of alcohol/Technovit 7200 VLC resin (Exakt Kulzer, Norderstedt), finally infiltrated, and later embedded in pure resin. One longitudinal cut in the core of each sample was performed in order to obtain two sections per tooth.

All obtained sections were glued with Technovit 7210 VLC resin (Exakt Kulzer, Norderstedt) on plastic slides, ground to a final thickness of 120 *μ*m. C and D teeth were stained with Toluidine Blue and Pyronin Y in order to visualize the soft tissue microstructure. All specimens were observed and microphotographs were acquired under transmitted and polarized microscope Nikon Eclipse 80i (Nikon, Japan), equipped with a digital camera at the magnification of 10x, 20x, 40x, and 60x.

### 2.1. Histological and Morphological Analysis

**Figure 5:**
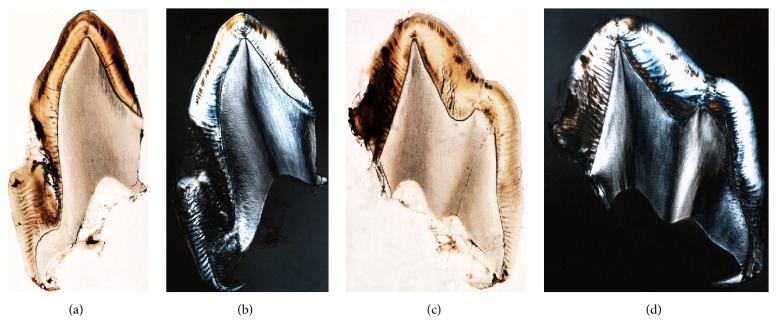
The ground sections of tooth A (a-b) and tooth B (c-d) under light microscope (a, c) and polarized microscope (b, d). The sections are not stained and polarization highlights mineralized tissue. Enamel appears more birefringent than dentine due to its higher level of calcification.

Histological examination revealed that the two supernumerary teeth A and B presented an altered architecture and a peculiar shape with the pulp cavity and the radicular portion missing. Ground sections and analysis by polarized light revealed well mineralized tissues, structurally resembling normal enamel and dentine. Morphological analysis suggests that tooth A (indicated in Figures 5(a) and 5(b)) presents a morphology similar to a canine tooth, while tooth B (indicated in Figures 5(c) and 5(d)) resembles a premolar tooth.

**Figure 6:**
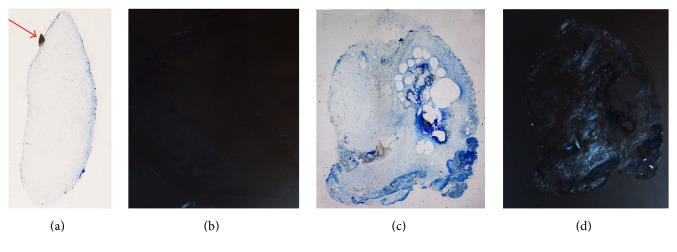
The ground sections of the two “ghost” teeth C (a-b) and D (c-d) under light microscope (a, c) and polarized microscope (b, d). The sections were stained with Toluidine Blue and Pyronin Y. Polarization highlighted a limited area containing mineralized tissue. The papilla in (b) did not present areas of calcified tissue, thus resulting as not birefringent.

In contrast, the other two “ghost” supernumerary teeth (C and D), which were discovered during surgery, could be compared to primordial dental germs and have resulted from an altered odontogenic process (Figures 6(a)–6(d)).

The tooth C showed a structure similar to a developing dental papilla characterized by dental pulp mesenchymal-like tissue (Figures 6(a) and 6(b)). The tissue was rich in blood vessels and the cells were characterized by a small cell body with a large, round nucleus and few long and thin cytoplasmic processes. Furthermore, at one point on the papilla border, indicated with a red arrow (Figure 6(a)), a small area of mineralized tissue similar to dentine was observed.

**Figure 7:**
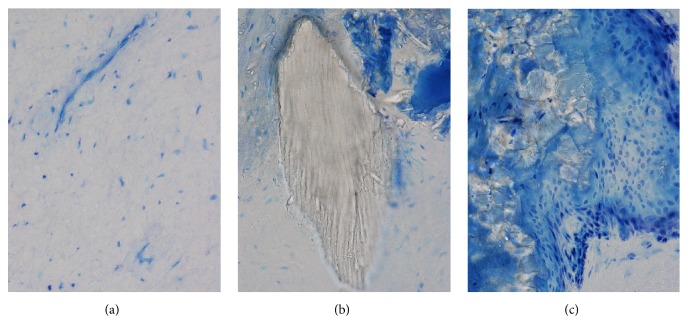
Details of the “ghost” teeth C and D under light microscope (a–c): (a) mesenchymal-like tissue; (b) dentine fragment; (c) odontoblast-like cells (red arrow) that appear to be in a phase of matrix deposition around calcified granules (yellow arrow). Toluidine Blue and Pyronin Y staining, total magnification 400x.

The tooth D presented an unusual structure and features. It was possible to distinguish a portion characterized by mineralized islands located mainly in peripheral area (Figures 7(a) and 7(b)) and mesenchymal-derived tissue similar to dental pulp core (Figure 7(c)).  Figure 7(a) shows one of the irregular dentine fragments, clearly identifiable by the presence of dentinal tubuli. The dentine fragments were present throughout the sample. In addition, the tooth contained numerous calcified granules not histologically structured and similar to primordial nuclei of mineralization. The presence of calcified tissue was further confirmed by the presence of birefringent areas revealed by polarized light.

Next to the granules, in the peripheral area, portions of dense connective tissue surrounded by cuboidal/cylindrical cells similar to preodontoblasts were present. The cells seemed to be in the process of creating typical fence/palisade structure and ready to deposit dentine (Figure 7(b), red arrow).

In the core of tooth D some follicles of dehydration due to histological processing were detected. They were characterized by different size and shape. Furthermore, the core was populated by many cells of mesenchymal origin similar to cells described in relation to tooth C (Figure 6(a)).

## 3. Discussion

The etiology of supernumerary teeth still remains unclear. Several theories have been proposed to explain their occurrence. The most common are phylogenetic theory; an abnormal reaction to a traumatic local event; environmental factors; the dichotomy of the tooth germ; and the theory of hyperactivity of the dental lamina [12]. The last one seems to be the more accepted [13, 14]. Heredity is considered to be an important factor, especially in the development of multiple supernumerary teeth, which are considered to represent a partial third dentition in humans. Their inheritance however does not seem to follow a Mendelian pattern [2, 12].

Supernumerary teeth may erupt normally or remain impacted and in both cases they can create problems because of their potential to interfere with normal occlusal development. They can cause crowding, delayed eruption of deciduous or permanent teeth, rotation of adjacent teeth, cystic formation, or migration of the unerupted teeth [2, 4, 10]. Based on morphology, supernumerary can be defined as supplemental (eumorphic) or rudimentary (conic shape, tuberculate, molariform, and odontome) [15, 16]. Seventy-five percent of the supernumerary teeth are asymptomatic [5] and are mostly diagnosed as an accidental radiological finding or because the presence of one of the anomalies seen before makes it necessary to get an OPT [12].

Surgical removal of the supernumerary teeth is indicated in case of delayed eruption, displacement of the adjacent tooth, cystic lesion, and resorption of the adjacent tooth, but if the risks of surgery are more than benefits, the teeth may be left in situ and monitored regularly [4].

**Figure 8:**
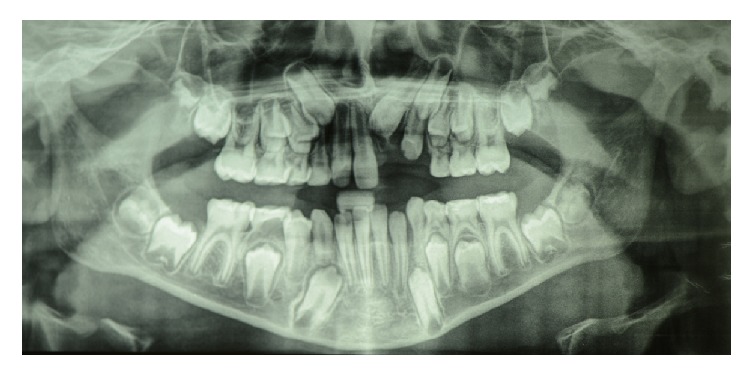
OPT of the patient during follow-up (1 year postextraction).

After supernumerary extraction, while monitoring with OPT (Figure 8), the patient of the present study will undergo a rapid maxillary expansion and then he surely will have to be treated with a fixed appliance.

Today, in addition to traditional OPT, CBCT is considered to be a great help in dental diagnosis about number, position, and morphology, because it allows more detailed information such as 3D reconstruction of teeth and bone with less X-rays than conventional computed tomography [10, 17, 18].

In the reported case, the supernumerary teeth were asymptomatic but caused the retention of 21. This is the reason why the patient was suggested to have an OPT. This first exam revealed the presence of two supernumerary teeth. An additional X-ray exam with a CBCT was requested in order to define how to plan a surgical intervention. Due to superimposing structural components, the correct diagnosis of the location, eruption patterns, and variations of supernumerary teeth is sometimes impossible with OPT and IOPA (intraoral periapical X-rays). The pediatric dental community can benefit from the amount of additional information provided by CBCT [19], though in this case the CBCT did not diagnose the two “ghost” elements.

This exam confirmed the presence of the first two “calcified” supernumerary teeth with rudimentary morphology. It was only during the extraction that the surgeon found another two “ghost” elements, likely remnants of an odontogenic cordon that interrupted proliferative process at different stages resulting in two dental abortive hints. “Ghost” teeth were not be seen in CBCT since they were not calcified enough. In fact element C resembled a dental papilla stopped at early stage while element D presented several granules of mineralized tissue typical of a later stage of proliferation/maturation.

The fragmentation of this eventual odontogenic cordon could lead to constituting other dental abortive elements either new or already formed but not detected. Therefore, this case seems to confirm the theory of hyperactivity of the dental lamina [8] recommending a careful clinical monitoring of the patient.

In fact, chronologically supernumerary teeth can develop simultaneously to permanent teeth or after them (after permanent dentition). Some authors described the formation of late supernumerary teeth [10, 20–23] and suggested clinical and radiographic monitoring in order to assess the formation of further teeth [4].

## 4. Conclusion

Supernumerary teeth have to be extracted when they could inhibit permanent teeth eruption. Appropriate diagnosis with OPT and CBCT is recommended and an accurate surgical site revision is mandatory. The orthodontic treatment should include the monitoring of the eruption before the disimpaction of the retained permanent tooth. This case report seems to confirm the existence of odontogenic hyperactive lamina in nonsyndromic patients that may alter the normal development or eruption of permanent teeth with eventual skeletal abnormalities during growth. This condition also suggests an accurate monitoring of patients with supernumerary teeth in order to discover any additional supernumerary element, already present at the time of the first examination but subjected to a later calcification, appearing in X-rays only after some time.
